# Discounting and Digit Ratio: Low 2D:4D Predicts Patience for a Sample of Females

**DOI:** 10.3389/fnbeh.2017.00257

**Published:** 2018-01-24

**Authors:** Diego Aycinena, Lucas Rentschler

**Affiliations:** ^1^Departament of Economics, Universidad del Rosario, Bogotá, Colombia; ^2^Department of Economics and Finance, Utah State University, Logan, UT, United States

**Keywords:** 2D:4D, digit ratio, time preferences, discounting, convex time budget, testosterone, economic experiments, economic behavior

## Abstract

Inter-temporal trade-offs are ubiquitous in human decision making. We study the relationship between preferences over such trade-offs and the ratio of the second digit to that of the forth (2D:4D), a marker for pre-natal exposure to sex hormones. Specifically, we study whether 2D:4D affects discounting. Our sample consists of 419 female participants of a Guatemalan conditional cash transfer program who take part in an experiment. Their choices in the convex time budget (CTB) experimental task allow us to make inferences regarding their patience (discounting), while controlling for present-biasedness and preference for smoothing consumption (utility curvature). We find that women with lower digit ratios tend to be more patient.

## 1. Introduction

Human decisions involving inter-temporal outcomes are ubiquitous. For example, decisions involving savings and consumption, investments in physical and human capital, and career and health choices all involve trade-offs across time. Economists and other social scientists typically study inter-temporal choices using models which parameterize how an individual weights consumption at different points in time. In particular, discounted utility models assume that individuals place a higher weight on consumption that is sooner; that is, individuals discount the future. Richer models allow for other factors that may also affect inter-temporal choices, such as utility curvature (i.e., the preference to smooth consumption over time), and present biasedness (i.e., higher discounting of the future if choices involve present outcomes)^1^.

Time preferences are heterogeneous among individuals (Harrison et al., 2002; Andreoni et al., 2015). That is, individuals vary in the degree to which they discount the future (their patience), in their preference to smooth consumption, and in their degree of present-biasedness. Given this heterogeneity and that the domain of inter-temporal preferences includes choices over important human capital decisions, it is not surprising that measures of discounting correlate with smoking, alcohol consumption addiction, and drug abuse (Kirby et al., 1999; Mitchell, 1999; Petry, 2001; Chabris et al., 2008; Sutter et al., 2013). In addition, Cadena and Keys (2015) finds that impatient individuals are more likely to make investments that can be classified as dynamically inconsistent and consequently end up with lower income on average. Golsteyn et al. (2014) finds that high discount rates have a negative relationship with school performance, labor supply, health and income. Kirby et al. (2002) also reports evidence of patience being positively correlated with literacy and schooling among the Tsimane' in Bolivia.

Thus, understanding the underlying determinants of inter-temporal preferences can help improve our understanding of human behavior over countless domains, as well as the welfare consequences thereof^2^. Indeed, we still know relatively little regarding the underlying determinants of inter-temporal preferences.

In this paper we examine whether a link exists between discounting and second-to-fourth digit length ratios (2D:4D)^3^. 2D:4D is a marker for pre-natal exposure to sex hormones (testosterone and estradiol) in males and females (Manning, 2002; Lutchmaya et al., 2004; Zheng and Cohn, 2011). Evidence suggests that exposure to sex hormones *in utero* has an organizational effect brain development (Goy and McEwen, 1980; Manning et al., 2001).

If exposure to sex hormones *in utero* has an effect on the brain, then examining a potential effect on time preferences seems warranted. Several studies find that higher cognitive ability is associated with more patience (Shamosh et al., 2008; Burks et al., 2009; Dohmen et al., 2010; Benjamin et al., 2013). Frederick (2005) introduced the cognitive reflection test (CRT), a simple test designed to capture the cognitive capacity to override an intuitive wrong answer and reflect upon the simple yet non-intuitive correct answer. High scores in this test correlate with higher cognitive abilities (as measured by the Wonderlic Personnel Test, the Need for Cognition Scale, etc.). Furthermore, Frederick finds that individuals with higher CRT scores are generally more patient (using hypothetical choices). In addition, Bosch-Domènech et al. (2014) reports that lower 2D:4D measures are associated with higher scores on the CRT. Collectively, these studies provide a rationale to examine the relationship between 2D:4D and discounting.

We use an experimental task, the convex time budget (CTB), to measure time preferences. This method has the advantage of allowing simultaneous structural estimation of discounting, utility curvature, and present-biasedness. The simultaneous estimation is important, as estimating them separately often results in estimates of discounting that are unrealistically high (Andersen et al., 2008).

External validity of time preferences measured via experimental tasks has been documented with different samples. Among school children, experimental measures of impatience are significant predictors of savings decisions, health behavior and school misconduct (Castillo et al., 2011; Sutter et al., 2013). Experimentally elicited present-biasedness is correlated with credit card debt among a sample of adults in Massachusetts (Meier and Sprenger, 2010), and predicts payments for environmental services in a sample of Ugandan farmers (Clot and Stanton, 2014). With the experimental task and sample reported here, (Aycinena et al., 2017) shows that preferences for consumption smoothing predict choices among a menu of payment options with large stakes.

The main contribution of this paper is to the literature on hormones and economic behavior. Specifically, we contribute to the literature that examines economic behavior and 2D:4D as a proxy for prenatal exposure to hormones and economic behavior (e.g., Brañas-Garza and Rustichini, 2011; Millet, 2011; Apicella et al., 2015). This literature has examined economic parameters such as risk preferences (Garbarino et al., 2011; Aycinena et al., 2014; Branas-Garza et al., in press), altruism (Branas-Garza et al., 2013; Galizzi and Nieboer, 2015), overconfidence regarding cognitive abilities (Neyse et al., 2016), etcetera.

There has been limited attention paid to the relationship between 2D:4D and time preferences. Drichoutis and Nayga (2015) uses two experimental tasks involving multiple price list to separately measure risk and time preferences and relates them to 2D:4D. Their evidence is mixed, but suggests that there may be a negative relationship between 2D:4D and discounting. Our paper differs in several important ways: first, they have a final sample of 138 (77 female) university students, while we have a sample size of 419 females who are not students. Second, we use five independent measures of 2D:4D taken from scans of our subjects hands using software designed for this purpose. This is intended to minimize measurement error, and increase the reliability of our measurements. Drichoutis and Nayga (2015) use rulers to measure 2D:4D, and did not scan the hands of their subjects. Third, they used the Holt and Laury (2002) method to measure risk aversion (which is presumed to measure utility curvature). This method involves subjects choosing between lotteries. We employ the CTB task, which does not involve choices over lotteries. Lucas and Koff (2010) analyzes the relationship between 2D:4D and delay discounting, but does not consider other parameters involved in inter-temporal choices (consumption smoothing and present-biasedness). They only find a significant relationship for the right hand for women. They find that a lower 2D:4D ratio is associated with greater delay discounting. Our paper differs significantly from this study in that we use a large sample of non-students, use a different elicitation method and jointly estimate multiple parameters underlying intertemporal preferences.

In addition to contributing to the hormones and economic behavior literature, this study also contributes to the economics literature exploring time preferences on three fronts. First, a robust correlation between time preferences and 2D:4D would provide an exogenous determinant of individual time preferences which could serve as an exogenous instrument to examine causal relations between time preferences and other economic behavior. This could be an important tool to examine causal relationships; for instance, in the growing literature exploring the link between patience and social preferences (Curry et al., 2008; Espín et al., 2012, 2015). Second, most economic theories implicitly or explicitly assume the stability of choice primitives (such as time and risk preferences) and there is empirical evidence of some stability in time preferences at the individual and aggregate levels (Kirby, 2009; Meier and Sprenger, 2015). The link between pre-natal exposure to hormones and time preferences suggests a (partial) mechanism through which time preferences can be heterogeneous across individuals and relatively stable over time. Finally, the third front links to the literature that shows that patience is correlated with higher cognitive ability (Shamosh et al., 2008; Burks et al., 2009; Dohmen et al., 2010; Benjamin et al., 2013). Given that cognitive ability seems to be correlated with 2D:4D (Brañas-Garza and Rustichini, 2011; Bosch-Domènech et al., 2014), our results may suggest a potential mechanism through which 2D:4D affects patience.

## 2. Materials and methods

*Acuerdo ministerial SP-M-466-2007* (regulating human clinical trials in Guatemala) did not apply to our study and no ethics committee has existed at our (former) institution in Guatemala. Nevertheless, we adhered to standard protocols involving studies that use experimental methods and measures of 2D:4D; specifically, no deception was used in the experiments, we obtained informed consent from participants, and we ensured privacy and security of data and decisions^4^.

### 2.1. Participants

Our sample consists of beneficiaries of Guatemala's Conditional Cash Transfer (CCT) program^5^. Due to CCT program requirements, our sample is 99.1% female and not representative for Guatemala^6^. As might be expected, relative to female respondents on a national representative survey, participants in our experiment are poorer, more likely to be or have been married, live in larger households and their living quarters are more precarious^7^.

After dropping some observations, the final sample in our analysis consists of 419 individuals^8^. These subjects reside in seven different municipalities across three departments: (El Progreso, Escuintla, and Sacatepéquez) where we ran experimental sessions. Ages range from 20 to 76 (mean 35.9, median 35). All of these women, as a condition for eligibility in the CCT program either have children or were pregnant at the time of the experiment.

### 2.2. Experiment

Participants performed several independent experimental tasks. The first and main task elicits inter-temporal choices using a version of the CTB introduced by Andreoni and Sprenger (2012a,b). The other tasks (which are not used in the current analysis) involve choosing how to spread receipt of financial windfall gains over time when there is no cost associated with receiving funds earlier, eliciting a subject's willingness to forgo funds in order to maintain intra-household control of a financial windfall, and/or a hypothetical CTB which elicited how subjects believed they would behave if questions were asked at a future date.

Participants earn an initial amount of *GTQ*50 (approximately USD6.4 or PPP$12.3) for taking part in the experiment^9^. In addition, they could earn between *GTQ*45 – *GTQ*100 (PPP$11.1 - PPP$24.7) based on their choices in the CTB. To put these amounts in context, CCT's entitled a household to receive GTQ150 (USD19.2 or PPP$37) per month, provided all household members comply with the conditions. Median self-reported household monthly income for the sample was in the range from GTQ500 to GTQ1,000 (PPP$123.5 to PPP$246.9) and 90% of participants report monthly household income below GTQ2,000 (USD256 or PPP$494).

#### 2.2.1. Convex time budget (CTB) task

In the CTB, participants see a series of 24 questions, knowing in advance that one of them will be randomly selected to determine their earnings. Each question presents a choice among six options that involve a combination of money to be obtained at two different times: *t* and *t* + *k* days after the experiment^10^. Implicit in the options was a trade-off between receiving money earlier (at time *t*) vs. delayed (time *t* + *k*): each of these 24 questions allowed subjects to eliminate the delay of partial amounts of money, by “transforming” delayed money (at time *t* + *k*) into early money (at time *t*) at a constant rate (marginal rate of transformation or MRT) that was weakly greater than one.

**Figure 1:**
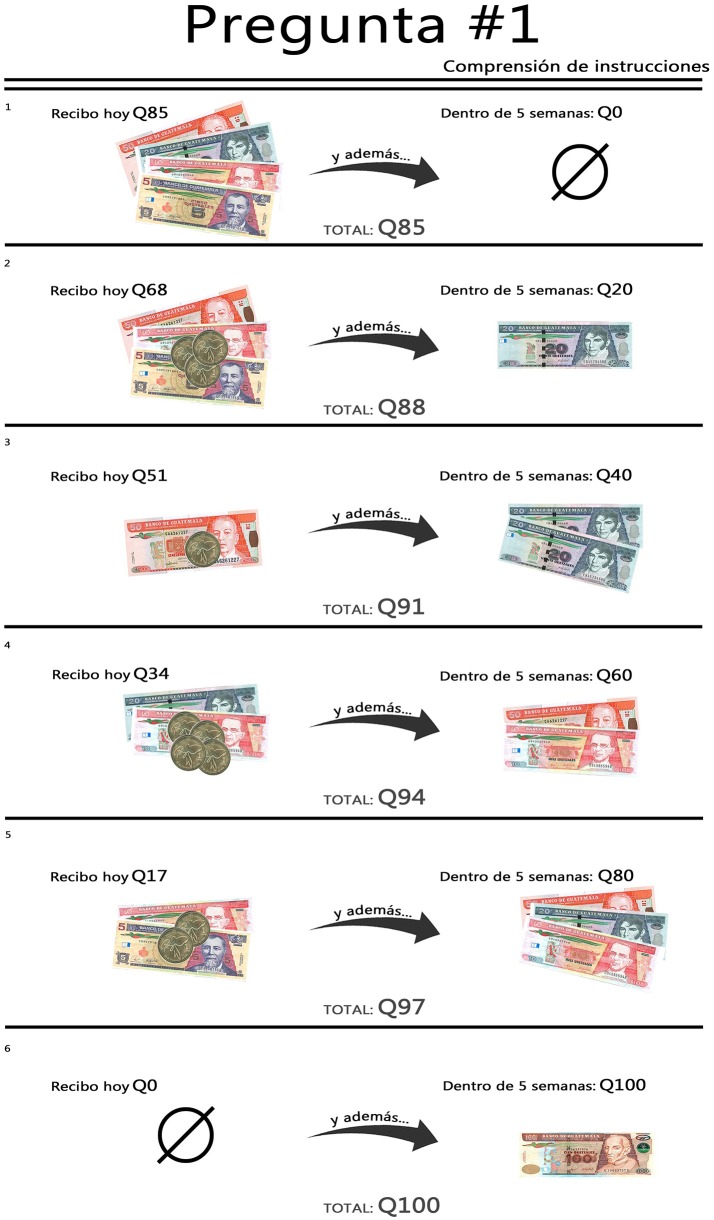
Example CTB question, as presented to participants.

More specifically, in each question, one option is GTQ100 at time *t* + *k*, and GTQ0 at time *t* (not including the split payments participation fee). Each of the remaining five options involve shifting GTQ20 from time *t* + *k* to time *t* at a constant marginal transformation rate (MRT) or relative price, until only GTQ0 remains at time *t* + *k*. Figure 1 illustrates the six options for a question (using *MRT* = 1.18, *t* = 0, and *k* = 35) as presented to participants^11^.

**Figure 2:**
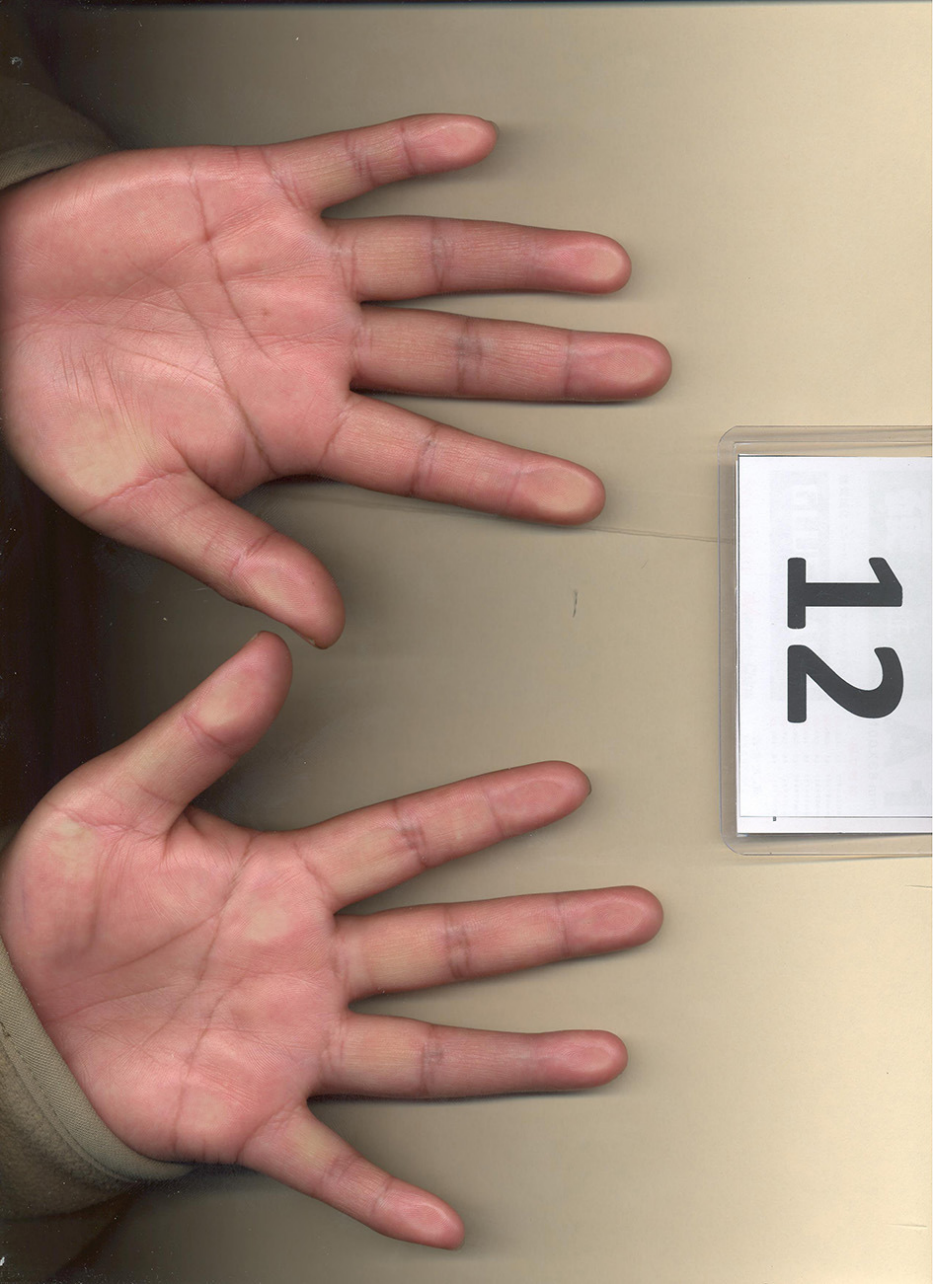
Example of hand scan image used to measure 2D:4D.

We used two values of *t*: *t* = {0, 35}. Each of these, were combined with two different delays: *k* = {35, 63}. The variation in the delay (*k*) allows inference regarding discounting of future utility, and the variation in the early period (*t* = 0 or *t* > 0) allows inference regarding present-biasedness. For each of the four combinations of *t* and *t* + *k*, participants are presented with six questions, each with a different MRT. As previously mentioned, each question presented six options to choose from. These include two options “at the corners” (all the money delayed or all early) and four options of “interior choices” (involving combinations of both, delayed and early money). The availability of interior choices allows inference regarding preferences for consumption smoothing (Aycinena et al., 2017). Table 1 summarizes the parameters used.

**Table 1:** Parameter summary for CTB task.

	**1**	**2**	**3**	**4**
***t***	**0**	**0**	**35**	**35**
***k***	**35**	**63**	**35**	**63**
MRT_1_	1.05	1.00	1.05	1.00
MRT_2_	1.11	1.05	1.11	1.05
MRT_3_	1.18	1.11	1.18	1.11
MRT_4_	1.25	1.33	1.25	1.33
MRT_5_	1.43	1.67	1.43	1.67
MRT_6_	1.82	2.22	1.82	2.22

Payments were implemented via post-dated checks made out to the participant. As in Andreoni et al. (2015), to guarantee that the transaction costs associated with obtaining the two associated payments are the same, the GTQ50 participation payment is evenly divided between the payment at time *t* and the payment at time *t* + *k*^12^.

We vary three things between experimental sessions to control for order effects. First, for each pair of *t* and *t* + *k*, we varied the order in which participants see the associated six questions. In some sessions the relative price of money at time *t* is decreasing over the six questions, and in other sessions it is increasing. We refer to this as the decreasing opportunity cost (DOC) treatment. Second, in some sessions the options within a given question are ordered such that the amount at time *t* is monotonically decreasing, and in other sessions it is increasing. We refer to this as the decreasing soon amount (DSA) treatment. Third, in some sessions, the GTQ25 payments for taking part in the experiment which was added to both the payment at time *t* and time *t* + *k* was explicitly shown in each question, and in others it was not. Note that this information was provided to participants prior to the CTB. This treatment simply varies the salience of the participation fee. We refer to this treatment as the included participation fee (IPF) treatment.

#### 2.2.2. Sessions and protocols

Experimental sessions took place in multipurpose rooms in the municipalities where subjects reside. We ran a total of 23 sessions with 16–24 subjects per session. Each session lasted between 3 and 4 h. All sessions were conducted by a session leader and a team of assistants.

Participants were asked to give informed consent upon arrival. After welcoming participants and giving a general introduction, the session leader projected at the front of the room and read aloud instructions for the CTB^13^. Afterwards, assistants ask each participant to answer several questions to ensure understanding. Then, assistants individually elicit answers for the first six questions (for *t* = 0 and *k* = 35, with MRT varying across questions). As noted above, since many participants are illiterate it was important for assistants to provide individual support and show decision sheets (illustrating the available options with pictures of the relevant monetary amounts) for each question. Once all participants have answered the first six questions the session leader explains the changes for the following six questions and assistants individually elicit participant responses. This process continues until all 24 questions of the CTB have been answered.

Once the CTB task is complete, the session leader reads instructions for the remaining tasks and the experiment continues until all experimental tasks are completed. Participants then got a short break where beverages and snacks were provided. A bingo cage was used to determine the question from the CTB task that would be paid. Assistants individually interviewed each participant for a socioeconomic survey. Participants were then called individually to receive their checks and sign receipts. At this time they were asked if we could scan their hands. If they consented to this, their hands were then scanned.

### 2.3. Digit ratio (2D:4D) measures

We collected scanned images of the participants' hands^14^. After all images were collected, a research assistant randomly divided the images into five batches^15^. Each batch contained a total of 108 images, including 10 re-inserted images from other batches (so that each rater measured the 2D:4D ratio for a total of 50 subjects twice). These repeated measures serve as the basis for assessing the consistency of measurement for each rater. Eight raters were instructed and received guidance on using the Autometric software (DeBruine, 2004) designed to measure digit ratios. They then independently measured both hands for each image in all five batches. The order in which each rater received the five batches was randomized.

Thus, we collected 8 independent 2D:4D measures for each hand of all participants. In addition, we had 50 randomly selected images measured twice by each rater. The repeated measures for the 50 randomly selected images allowed us to measure intra-rater consistency of 2D:4D measures. We drop the measures for three raters with an intraclass correlation coefficient (ICC) < 0.85. This leaves us with five high quality measures for each hand of each participant. The ICC for the repeated measures of the remaining raters range from 0.8625 to 0.9772 and the Spearman ρ range from 0.8548 to 0.9754. In no case are there statistically significant differences in the means of the repeated measures. Table 2 shows measures of intra-rater consistency.

**Table 2:** Within-rater consistency using repeated measures (for both hands).

	**Left hand**			**Right hand**		
	**ICC**	**Rho**	***p*-value**	**ICC**	**Rho**	***p*-value**
Rater 1	0.977	0.975	0.213	0.945	0.937	0.792
Rater 2	0.925	0.913	0.398	0.962	0.946	0.301
Rater 3	0.940	0.945	0.181	0.945	0.921	0.952
Rater 4	0.908	0.904	0.506	0.915	0.903	0.417
Rater 5	0.876	0.855	0.569	0.863	0.886	0.334

*Within-rater analysis of repeated measures. Table contains intra-class correlation coefficient (ICC), Spearman's rho correlation coefficients (Rho), and p-value for two-sided paired t-test for equality of means between raters measures for left and right hands, correspondingly*.

Table 3 displays the between-rater correlation coefficients. Between rater correlation coefficients range from 0.8663 to 0.9392 for the right hand measures, and from 0.7546 to 0.9668 for the left hand.

**Table 3:** Correlation coefficients for between-rater measures for left-hand and right-hand measures.

	**Left hand**					**Right hand**				
	**Rater 1**	**Rater 2**	**Rater 3**	**Rater 4**	**Rater 5**	**Rater 1**	**Rater 2**	**Rater 3**	**Rater 4**	**Rater 5**
Rater 1	1.000					1.000				
Rater 2	0.897	1.000				0.880	1.000			
Rater 3	0.906	0.928	1.000			0.967	0.923	1.000		
Rater 4	0.939	0.858	0.899	1.000		0.956	0.872	0.930	1.000
Rater 5	0.866	0.872	0.880	0.882	1.000	0.803	0.755	0.820	0.773	1.000

*Table contains Spearman rho correlation coefficients between raters measures for left and right hands, respectively*.

**Figure 3:**
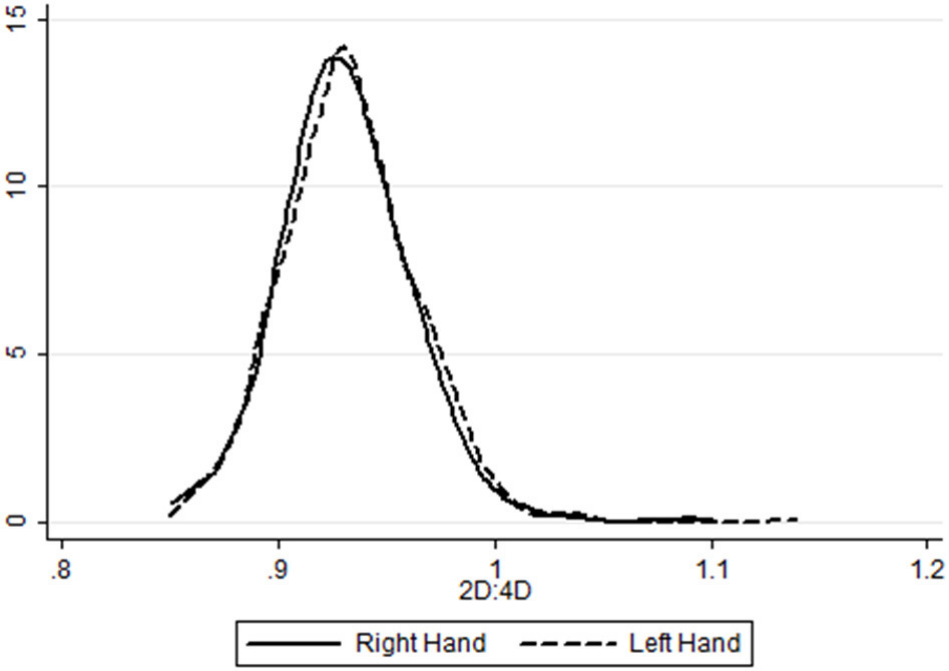
Kernel densities of 2D:4D measures.

We take the average across the five measures^16^. Table 4 shows the summary statistics for the 2D:4D measures. The digit ratios for our sample are lower than those typically found in the literature. For the right hand, mean 2D:4D is 0.9322 (with a standard deviation of 0.0315); for the left hand the mean is 0.9337 (with a standard deviation of 0.0321)^17^. No statistical significant difference is found in variance or mean between hands. Figure 3 illustrates the distribution of the average of all five measures for both hands.

**Table 4:** Summary statistics of the 2D:4D ratio.

	**Left hand**	**Right hand**
Mean	0.933	0.931
Median	0.931	0.930
Standard deviation	0.032	0.032
Min	0.8492	0.8508
Max	1.1396	1.1006

Thus, our final 2D:4D data consists of the average of five (high quality) independent measures for the 419 final sample subjects.

## 3. Results

### 3.1. Plan of analysis

Given the so called “replicability crisis” in scientific findings (see e.g., Ioannidis, 2005; Button et al., 2013; Aarts et al., 2015; Camerer et al., 2016), we attempted to limit the degrees of freedom available to us as researchers^18^.

To limit the degrees available to us, we partnered with Anna Dreber to prepare an analysis plan^19^. In the plan, we specify that our main method of analysis will rely on the interval censored Tobit model to structurally estimate time-preference primitives, which allow discounting to vary with 2D:4D. Specifically, we estimate discounting (δ) as a linear function of 2D:4D (among other parameters).

In the analysis plan we also specify three robustness tests. First, we test robustness to changes in the background parameters, since (Andreoni et al., 2015) and (Aycinena et al., 2017) show that the structural estimates may be sensitive to whether or not the participation fee (among other background parameters) is included in the analysis. Thus we perform two robustness checks which modify assumptions about the background parameters.

Second, we examine whether the results are robust at the individual level. To do so, we structurally estimate time-preference primitives at the individual level, and test whether the individual level estimates for δ are correlated with the individual 2D:4D measures. Finally, our third robustness check tests whether our results depend on the method of structural estimation. To do so, we drop the structural estimation approach and test whether 2D:4D measures predict choices of more delayed money using reduced form analysis.

### 3.2. Theoretical and econometric framework

To analyze choices, we rely on a model inter-temporal preferences that assumes a time-separable quasi-hyperbolic utility function with constant relative risk aversion. Specifically, denoting the amount of money received by subject *i* at time *t* (*t* + *k*) as *x*_*it*_ (*x*_*it* + *k*_), we assume that the following utility function underlies observed choices:

(1) $$U\left(x_{it},x_{it+k}\right)=\{\begin{array}{ll} x_{it}^{\alpha }+\beta \delta^{k}x_{it+k}^{\alpha } & \text{ if }t=0\\ x_{it}^{\alpha }+\delta^{k}x_{it+k}^{\alpha } & \text{ if }t>0. \end{array}$$

Our framework includes three parameters that affect time-preferences: discounting (δ), present biasedness (β) and utility curvature (α). The discount factor, δ, captures the degree to which an individual discounts delays in consumption. A δ = 1 implies that individuals are so patient, that all else equal, they are indifferent to delays in consumption. The lower the value of δ (δ < 1) implies higher discounting of delaying consumption, that is, less patience. Present biasedness, β < 1, captures how much (more) an individual discounts delaying consumption relative to immediate consumption. Note that β = 1 implies a standard discounting model with no present biasedness. Finally α, utility curvature, underlies preferences to inter-temporally smooth consumption. An α = 1 implies that consumption is perfectly substitutable across time, thus no preference to smooth consumption in time. The lower the value of α (α < 1) the higher the preference to smooth consumption. That is, all else equal, the lower α, the more an individual is willing to sacrifice in order to attain a consumption profile that is smoother across time.

Notice that these three parameters are interrelated for time-preferences. That is, it is possible to observe the same choice by two individuals with very different levels of patience (different δ's) if there utility curvature (α) and/or present-biasedness (β) also differ. Given this, it is important to estimate these three parameters jointly (see e.g., Andersen et al., 2008; Andreoni and Sprenger, 2012a).

### 3.3. Main analysis: structural estimation

In our main analysis we employ interval censored tobit regressions^20^. This procedure jointly estimates three parameters: α, β, and δ.

The parameter δ is the aggregate measure of the time preferences in the population (see Andreoni et al., 2015 for a detailed description of the model and the estimation techniques). To test our hypothesis, we allow δ to be a function of the 2D:4D ratio. As specified in our analysis plan, the functional form we assume is as follow:

(2) $$\delta_{i}=\rho_{0}+\rho_{1}\cdot 2D:4D_{i}+\rho_{2}\cdot DSA_{i}+\rho_{3}\cdot DOC_{i}+\rho_{4}\cdot IPF_{i}$$

where experimental treatments [included participation fee (IPF) explicitly treatment, decreasing opportunity cost (DOC) treatment, decreasing soon amount (DSA) treatment] are included to control for differences in how the CTB task was presented to subjects.

The first two columns of Table 5, estimated separately, present results of the parameter estimates for the left and right hands of participants. The value for the parameter α shows a strong preference for smoothing consumption over time. The β parameter is higher than one, thus it shows no evidence of present-biasedness^21^. Next we present results in which the parameter of interest, δ, is a function of 2D:4D and treatment controls.

**Table 5:** Parameter estimates.

	**Main estimates**		**Robustness check 1.1**		**Robustness check 1.2**	
	**Left hand**	**Right hand**	**Left hand**	**Right hand**	**Left hand**	**Right hand**
α	0.540^***^	0.540^***^	0.727^***^	0.727^***^	0.877^***^	0.877^***^
	(0.017)	(0.017)	(0.010)	(0.010)	(0.005)	(0.005)
β	1.105^***^	1.105^***^	1.096^***^	1.096^***^	1.111^***^	1.111^***^
	(0.017)	(0.017)	(0.016)	(0.016)	(0.020)	(0.020)
ρ_0_ (*Constant*)	9.778^***^	13.524^***^	9.150^***^	12.645^***^	11.665^***^	16.021^***^
	(1.630)	(1.687)	(1.530)	(1.582)	(2.039)	(2.084)
ρ_1_ (2*D*:4*D*_*i*_)	−11.899^***^	−15.959^***^	−12.688^***^	−14.974^***^	−14.684^***^	−19.404^***^
	(1.738)	(1.800)	(1.632)	(1.690)	(2.181)	(2.235)
ρ_2_ (*DSA*_*i*_)	0.391^***^	0.361^**^	0.359^***^	0.331^**^	0.411^***^	0.375^***^
	(0.111)	(0.110)	(0.104)	(0.104)	(0.131)	(0.130)
ρ_3_ (*DOC*_*i*_)	0.159	0.186^+^	0.146	0.171	0.333^**^	0.361^***^
	(0.111)	(0.111)	(0.105)	(0.104)	(0.132)	(0.132)
ρ_4_ (*IPF*_*i*_)	1.073^***^	1.094^***^	1.006^***^	1.026^***^	1.468^***^	1.495^***^
	(0.114)	(0.114)	(0.107)	(0.107)	(0.133)	(0.134)
σ	1.521^***^	1.518^***^	2.411^***^	2.406^***^	6.639^***^	6.621^***^
	(0.040)	(0.040)	(0.065)	(0.064)	(0.117)	(0.117)
Log-likelihood	16,235.6	16,226.1	16,204.9	16,186.7	18,480.4	18,461.6
BIC	−32,351.4	−32,332.5	−32,290.1	−32,253.7	−36,841.1	−36,803.5

*Robust standard errors are reported in parenthesis*.

+ p < 0.1,

^*^ p < 0.05,

** p < 0.01,

*** *p < 0.001*.

For the parametrization of the discount factor (δ), we see that the coefficient on 2D:4D is negative (−11.899 for the left hand and −15.959 for the right hand) and statistically significant for both hands at the 0.001 level. This implies that lower 2D:4D is correlated with a higher discount factor. That is, individuals with lower 2D:4D (a marker for higher exposure to testosterone *in utero*) make more patient choices.

Following our analysis plan, we also explore whether there is evidence of a non-linear effect of 2D:4D on discounting. Specifically, we examine whether there is a quadratic relationship by adding 2D:4D^2^ as an explanatory variable. Under this specification (not reported but available from the authors upon request), we find that both the linear and squared coefficients are negative, but none are statistically significant at conventional levels.

## 4. Robustness checks

### 4.1. Robustness to changes in background parameters

It should be noted that the previous parameter estimates may be sensitive to whether or not the participation fee, among other background parameters, is included (e.g., Andreoni et al., 2015; Aycinena et al., 2017). Since all subjects received the participation fee, we included it (Q50, split evenly across two time periods) as a background parameter in the estimates reported in the previous section. For our first set of robustness checks, we test how sensitive our results are to modifying the background parameters.

We examine two alternative specifications of the background parameters. Our first examination involves dropping the participation fee from our analysis, so that *x*_*it*_ and *x*_*it* + *k*_ do not include the participation fee in our econometric analysis. We report the results for left and right hand in columns 3 and 4 of Table 5 (under the heading “Robustness check 1.1”). For the second, we estimate the parameters with the explicit option displayed to participants, according to the IPF treatment^22^. The last two columns of Table 5 (under the heading “Robustness check 1.2”) report the results of such estimates.

As the table shows, estimates of α seem to be quite sensitive to the background parameters used. The estimate of β on the other hand, seems quite robust. Regarding our coefficient of interest, although not quite as sensitive as α, δ does vary with the background parameters employed. Although the impact is not obvious due to the five parameters involved in the estimation of δ, the mean value of δ ranges from 0.6 to 0.85.

Nevertheless, the point to note is that the coefficient on 2D:4D is negative and statistically significant (*p* < 0.001) for both hands across all specifications. Thus, the relationship between 2D:4D and patience reported in the previous section seems robust to the specification of the background parameters.

### 4.2. Individual level estimates

The second robustness check involves attempting to estimate time preference primitives at the individual level. We use the interval censored Tobit model with 24 observations per individual (one observation for each of the 24 questions of the CTB) and attempt to jointly estimate α, β, and δ.

Unfortunately, our individual estimates are very imprecise. For our parameter of interest, δ, values range from 0 to 1.4*e*^191^, and the distribution is very skewed with a mean of 3.4*e*^188^, and for over half of the observations the estimate of δ < 0.0001.^23^ This lack of precision is not surprising given that for each individual, we have 24 observations to estimate eight parameters^24^. To try to overcome this problem, we restrict our analysis to individuals with an (arbitrarily defined) sensible δ parameter: individuals with 0 < δ < 2. This reduces drastically our subsample to 168 individuals.

We use the parameter estimates for the 168 individuals of our restricted sub-sample as a dependent variable and estimate the following reduced form model (separately for left and right hands) using OLS:

(3) $$\delta_{i}=\rho_{0}+\rho_{1}2D:4D_{i}+\rho_{2}\cdot DSA_{i}+\rho_{3}\cdot DOC_{i}+\rho_{4}\cdot IPF_{i}+\epsilon_{i}$$

We present results in the first two columns of Table 6. For the sake of brevity, we only present the results for 2D:4D (point estimate of ρ_1_ and its standard error) and the adjusted *R*^2^. The top row presents the 2D:4D coefficient for the left hand and the bottom row for the right hand, each estimated independently. None of the coefficients are statistically significant. The signs of the coefficients are consistent with our main analysis, except for the left hand when we include session and surveyor fixed effects. The adjusted *R*^2^ is negative for all four specifications of robustness check two, which indicates that the model is a very poor fit for the data^25^. Overall, this suggests that this approach was not successful in allowing us to test the robustness of the results^26^.

**Table 6:** Reduced form analysis robustness checks.

	**Robustness check 2**		**Robustness check 3.1**		**Robustness check 3.2**	
	**(1)**	**(2)**	**(1)**	**(2)**	**(1)**	**(2)**
Left hand	−0.039	0.390	−2.608^**^	−1.767^+^	69.401^**^	44.125^+^
	(0.332)	(0.332)	(1.515)	(1.358)	(25.154)	(22.902)
Adjusted *R*^2^/Pseudo *R*^2^	−0.019	−0.005	0.021	0.041	0.217	0.265
Right hand	−0.305	−0.140	−3.396^**^	−2.173^*^	94.875^**^	57.113^*^
	(0.338)	(0.382)	(1.609)	(1.423)	(27.502)	(24.925)
Adjusted *R*^2^/Pseudo *R*^2^	−0.017	−0.008	0.022	0.042	0.221	0.266
Observations	168	168	10,053	10,053	10,053	10,053
Session fixed effects?	No	Yes	No	Yes	No	Yes
Surveyor fixed effects?	No	Yes	No	Yes	No	Yes

*Point estimates for 2D:4D coefficient of the robustness checks. Robust standard errors are reported in parenthesis (clustered at the individual level for robustness checks 3.1 and 3.2) Robustness checks 2 and 3.2 are estimated using OLS; adjusted R^2^ for each hand is reported below standard errors. Robustness check 3.1 is estimated using ordered probits; Pseudo R^2^ is reported below the standard errors*.

+ *p < 0.10*,

* *p < 0.05*,

** *p < 0.01*,

*** p < 0.001.

### 4.3. Reduced form analysis

In our third robustness check, we bypass the structural estimation and directly examine choices with a reduced form approach. The independent variables we employ include our variable of interest (2D:4D), the marginal rate of transformation for the question (*MRT*_*j*_), the time when the early amount is to be received (*t*_*j*_), the delay (*k*_*j*_), and controls for our three treatment variables (*DSA*, *DOC*, *IPF*). Since we have multiple observations per individual, we cluster standard errors at the individual level. In all of our reduced form analysis, we estimate the model for both right and left hand 2D:4D.

Since participants could choose among six discrete ordered options (*Y*_*ij*_ ∈ [1, 2, …., 6]), we first examine this using an ordered probit model. Choosing option 1 maximizes the amount received in the early payment; choosing option 6 maximizes the amount received in the delayed payment. Thus, all else equal, a more impatient individual (i.e., with a lower δ) will tend to select lower options than a more patient individual (someone with a with higher δ). If our results are robust, we would again expect a negative coefficient for 2D:4D.

We present the results (of our coefficients of interest) in the middle columns (Robustness check 3.1) of Table 6. Column (1) presents the coefficients for the model described above. We find that for both hands, coefficients are negative and statistically significant (*p* < 0.01). Again, this supports the findings from the main estimates that lower 2D:4D individuals make more patient choices. Column (2) adds session and surveyor fixed effects. Under this specification, the coefficient for the left hand is no longer statistically significant at conventional levels (*p* < 0.1).

For our second reduced form approach, we use ordinary least squares and the dependent variable is the early amount chosen (*x*_*ijt*_) by individual *i* in question *j*. We use the same independent variables, with our focus again being on the coefficient of the 2D:4D^27^. Notice the the higher the early amount chosen, the more impatient the individual (given the tradeoffs between early and delayed amounts). Thus, in this approach, we expect a positive correlation between 2D:4D and our dependent variable.

Results for our coefficients of interest are reported in the last two columns (Robustness check 3.2) of Table 6. For the first specification (Column 1), the coefficients for both hands are positive and statistically significant (*p* < 0.01). In column (2) we add session and surveyor fixed effects. In this case, the coefficient for the left hand is no longer statistically significant at conventional levels (*p* < 0.1).

Again following our analysis plan, we perform an exploratory analysis of whether the relationship between 2D:4D and discounting is non-linear by adding 2D:4D^2^ as an explanatory variable. We do not find any robust evidence for a non-linear relationship between 2D:4D and discounting. Coefficients are not statistically significant either in the ordered probit or the OLS model.

To summarize this last robustness test, we find that results do not depend crucially on the assumption and methods of the structural estimation. Using reduced form analysis, we find evidence that 2D:4D is negatively related to patience for both hands in the first specification, and for the right hand in the second.

## 5. Discussion

In this study we investigate the impact of 2D:4D, as a proxy for pre-natal exposure to testosterone, on discounting. We use a large sample (*N* = 419) of low income females from a wide age range. We rely on 24 choices per individual using the convex-time budget task with large stakes, and the average of five independent measures of 2D:4D.

We follow an analysis plan and jointly estimate time preference parameters and the curvature of the utility function, and allow the discount parameter (δ) to to vary with 2D:4D. We find that, for both hands, 2D:4D is negatively correlated with discount factor (*p* < 0.001). That is, we find that lower 2D:4D generates more patient choices.

We stick to our analysis plan and perform three robustness tests. First, we examine robustness of our results to varying background parameters; and find that our results are robust. Next, we attempt to estimate time-perference parameters at the individual level and correlate them with 2D:4D using reduced form models. Results of this second robustness check are mixed, since our individual level parameter estimates are very noisy. Our third robustness test involves replacing the parametric estimation method with a direct reduced form analysis. For each hand we run two tests using ordered probits and two using OLS. Given the criteria pre-specified in our analysis plan, our results are mixed. We pre-defined that we would consider a result to be significant if *p*−*value* < 0.05 for both hands^28^. Specification (1) of robustness checks 3.1 and 3.2 satisfies this criteria. However, for specification (2), only the result for the right hand is significant at *p* < 0.05.

Our result are in contrast to those of Lucas and Koff (2010), which reports that lower digit ratios are correlated with greater discounting among women. Our findings also differ from those of Drichoutis and Nayga (2015), which report no effect of digit ratio on (risk or) time preferences. These differences might stem from different samples, methods or protocols used.

However, our finding that lower 2D:4D leads to more patience is consistent with the combined results from other studies that relate 2D:4D, cognitive ability and patience. Bosch-Domènech et al. (2014) find that lower 2D:4D is associated with higher scores in the cognitive reflection test (CRT), and Frederick (2005) finds that higher CRT scores correlate with more patience (in hypothetical choices) and with higher cognitive abilities^29^ These results are also consistent with other studies which also find that higher cognitive ability is associated with more patience (Shamosh et al., 2008; Burks et al., 2009; Dohmen et al., 2010; Benjamin et al., 2013).

Why should we care about the relationship between 2D:4D and discounting? Time preferences, and discounting in particular, play an important role in human decision making over countless domains (health, human capital accumulation, labor supply, income, etc.) with important welfare consequences. Our results are thus important, as they point to a potential biological underpinning of time preferences.

On a more methodological note, this finding suggests an exogenous determinant of individual time preferences. This may have broad implications for economic studies on the causal effect of time preferences on different economic behavior. That is, our results could be an important advance in identification strategies for researchers seeking to identify causal relationships between time preferences and other economic behavior, by using 2D:4D as an exogenous instrument.

This study has several peculiarities. First, our sample also differs from typical 2D:4D samples, as we do not rely on a WEIRD (Western Educated Industrialized Rich Democratic) population sample (Henrich et al., 2010a,b). Rather, our sample is particular on different margins: low income non-Caucasian females enrolled in a conditional cash transfer program. In addition, the 2D:4D measures of our sample are lower than those typically found in the literature. As with most findings, our results should be replicated to improve our confidence in the findings (Maniadis et al., 2017). In particular, this work should be replicated with samples of men. One limitation of this study is that our sample is exclusively female. As Frederick (2005) noted, there is a higher correlation of time preferences with CRT for females than males.

## Author contributions

DA coordinated the study, designed the experiment, coordinated 2D:4D measurements, conducted statistical analysis and drafted the manuscript. LR coordinated the study, designed the experiment, conducted statistical analysis and drafted the manuscript. All authors gave final approval for publication.

### Conflict of interest statement

The authors declare that the research was conducted in the absence of any commercial or financial relationships that could be construed as a potential conflict of interest. The reviewers AME and BL and the handling Editor declared their shared affiliation.

## Footnotes

^1^Discounting measures how much more a subject values consumption at an earlier date relative to a delayed later date. Present-biasedness refers to an increase in discounting when the earlier date under consideration is the present. See e.g., Laibson (1997); O'Donoghue and Rabin (1999).

^2^Several papers attempt to explore the covariates of time preferences (Lawrance, 1991; Pender, 1996; Harrison et al., 2002; Tanaka et al., 2010; Cassar et al., 2017). However, establishing a causal effect between the covariates and time preferences has proven to be challenging. For instance, Carvalho et al. (2016) attempts to explore the impact of poverty or lack of liquidity on discounting.

^3^The null hypothesis is that no correlation exists. As specified in our registered analysis plan, our alternative hypothesis is that 2*D*:4*D* is negatively correlated with patience; that is, low digit ratio is related to a higher degree of patience.

^4^Given the anticipated low levels of schooling and literacy, assistants read the informed consent sheet to each individual, marked whether subjects gave informed oral consent, and signed the sheet.

^5^*Mi Bono Seguro* (My Security Bonus) is a targeted CCT program overseen by the *Ministerio de Desarrollo Social* (Ministry of Social Development) of Guatemala. It aims to improve human capital accumulation by promoting investments in health and education for poor households with pregnant women or children under the age of 16.

^6^As is conventional among CCT programs, females tend to be the recipients of the funds. This program uses geographic targeting and proxy means testing for eligibility. This program offers two types of conditional transfers: an education transfer and a health transfer. To obtain the health transfer all children under 15, and all pregnant or breastfeeding woman must attend regular medical check-ups. To obtain the education transfer all children between the ages of 6 and 15 must have a school attendance rate of at least 90%. Households may be eligible for both transfers.

^7^We compared our sample with the 2011 National Survey of Living Conditions (ENCOVI). ENCOVI is a national representative household survey focused on the measurement of living standards run by the National Institute of Statistics (INE) of Guatemala. To maximize comparability, we restricted attention to female ENCOVI respondents in a comparable age bracket. For detailed results of this comparison, see Aycinena et al. (2015). Not surprisingly, there are limitations with the comparison between our sample and the ENCOVI data. ENCOVI is a national representative survey that was implemented between March and August of 2011, 2 years before our field work began. This was, however, the closest LSM household data set available from INE.

^8^We dropped 4 men, 29 participants who showed no variation across all 24 choices, 36 potentially questionable observations (based on inconsistencies between the metadata in the image files and the session data), 1 individual for whom there is no consent form, and 2 individuals who refused to have their hands scanned.

^9^Guatemala's local currency is the Quetzal (GTQ). According to Guatemala's Central Bank, the average market exchange rate for the relevant period was GTQ7.8177 per USD. For 2013, World Development Indicators PPP conversion factor for private consumption was GTQ4.0499 per international dollar at purchasing power parity (PPP$).

^10^In the parlance of economics, each question presents six points uniformly distributed along an inter-temporal budget constraint regarding money at time *t* and at time *t* + *k*.

^11^Since participants have low levels of literacy and numeracy, we presented all choices in the CTB using both numbers, and pictures of the associated quantities of money. Notice that each option specified the amount at time *t* and the amount at time *t* + *k*; as well as the total amount. To further ensure that participants understood the task, assistants asked each participant the questions individually, resolved any questions as they arose and recorded the participant's decision.

^12^During the implementation there was a problem with the post-dated check payment mechanism, as some participants were able to cash checks earlier than the dates indicated on them. This would be problematic for our parameter estimates if participants *anticipated* that this was a possibility, as their effective MRT would then be equal to one in all cases. More specifically, if participants *anticipated* this, then we would expect that they would choose the option that would allow them to maximize the total amount of money over early and delayed payments. As long as the experimental MRT was greater than one, they would choose the option with the minimum early payment and maximum delayed payment. However, this is not what we observe. Reduced form regressions on early check cashing find no statistically significant correlation between cashing checks early and choosing options that concentrate amounts on delayed payments. Results are available upon request.

^13^The supplementary material shows the text of the instructions for both experimental tasks, translated from the original Spanish.

^14^Using a digital scanner is a common method for taking digit ratio measures that has been shown to be reliable (Kemper and Schwerdtfeger, 2009). An example of a scan can be seen in Figure 2.

^15^We split the measurement of images into batches to break the task into smaller sub-tasks, in an attempt to reduce the effects of fatigue or boredom for research assistants measuring the digit ratios.

^16^Voracek et al. (2007) suggests using the average of multiple independent measures by different raters.

^17^Dropping the highest and lowest (to mitigate the potential impact of outliers) and taking the average of three intermediate measures, we would have for the right hand a mean of 0.9323 (with a standard deviation of 0.0316), and for the left hand a mean of 0.9330 (with a standard deviation of 0.0322). Other samples tend to report higher 2D:4D measures; for instance Branas-Garza et al. (in press) reports mean of 0.9734 and 0.9775 for female left and right hands. Aycinena et al. (2014) reports a mean of 0.957 and 0.954 for female left and right hands. This difference might be due to the different ethnic compositions of the different samples.

^18^Studies may give researchers many degrees of freedom, even without explicit fishing (Gelman and Loken, 2014). In 2D:4D research this problem is not absent; if anything it may be exacerbated as there is no consensus regarding which hand to use, which measures (mean, median, etc.) to use, or the correct specification (linear, quadratic, etc.) to employ.

^19^We thank Anna Dreber for her time helping us prepare the analysis plan while she was blind to the data. The plan is posted at the Open Science Framework web platform: https://osf.io/ey67f/register/564d31db8c5e4a7c9694b2be. It should be noted that, technically, this is not a pre-analysis plan, since we developed it after data collection was finished. Nevertheless, we feel that by developing it jointly with a credible third party, it helps to reduce the degrees of freedom of our analysis.

^20^For the structural estimation, the covariance matrix was estimated using sandwhich estimator for robust standard errors. See Aycinena et al. (2014) for a detailed description of the estimation method.

^21^Balakrishnan et al. (2017) suggests that present biasedness is only existent when payments are “truly immediate.”

^22^Recall that in this treatment, some subjects were shown amounts in the CTB that explicitly included the participation fee, while others were shown amounts that did not include the participation fee.

^23^The 25th percentile is zero, with a mean of 3.4*e*^188^ and median of .00001.

^24^The three parameters which measure preference primitives (α, β, and δ), in addition to the auxiliary parameters (σ, and the five cut-offs λ_1_, λ_2_, λ_3_, λ_4_, λ_5_).

^25^It should be noted that this is not driven by the 2D:4D measure, as a model that excludes 2D:4D as an explanatory variable also has negative adjusted *R*^2^ of similar magnitude. More importantly, the partial *R*^2^ (or coefficient of partial determination) of the 2D:4D coefficient is always positive, suggesting that if anything, it helps the model fit of the data (although clearly not enough).

^26^Although our attempt to estimate parameters at the individual level failed, we believe important to stick to our analysis plan and report the attempt despite its failure.

^27^It should be noted that the analysis plan specified that the dependent variable for this approach would be the delayed amount chosen. That is a mistake, since the delayed amount is a linear transformation of the dependent variable used in the first approach (Robustness check 3.1). Results are qualitatively and statistically the same if we use delayed amount as our dependent variable.

^28^The analysis plan states: “Since we will look at the correlation between discounting and 2D:4D for both hands, there is concern about multiple testing. We will consider a result to be significant if the *p*-values corresponding to the coefficients of 2D:4D for both hands are <0.05.”

^29^It should be noted that in Bosch-Domènech et al. (2014), 2D:4D still predicts CRT scores even after controlling for (hypothetical) patience).
