# Supplementary material for: Discounting and Digit Ratio: Low 2D:4D Predicts Patience for a Sample of Females
Source: Front Behav Neurosci. 2018 Jan 24;11:257. doi: 10.3389/fnbeh.2017.00257 (PMC5788003; doi:10.3389/fnbeh.2017.00257)
Supplement: Supplementary file 1 [file DataSheet1.pdf]

Supplementary Materials for “Discounting and digit ratio: Low 2D:4D predicts patience for a sample of females”

Diego Aycinena [1,*] **and Lucas Rentschler** [2]

[1] *Departamento de Economía, Universidad del Rosario, Bogotá, Colombia*
[2] *Department of Economics and Finance, Utah State University, Logan, Utah*

Correspondence*:
Casa Pedro Fermín, Calle 12C No. 4-59 - Bogotá D.C. Colombia
diego.aycinena@urosario.edu.co

1 INSTRUCTIONS

- Now, we will start the experiment. The rules of the experiment are:
  1. You can not talk to any participant of the session.
  2. You can not use cellphones.
  3. All the data and answers that you provide to us will be totally CONFIDENTIAL.
- The experiment consists of three parts.
  1. By participating in and completing the three parts of the experiment, you will receive $Q50.00$. (These $Q50.00$ will be divided in two payments).
  2. In addition, you can also earn additional money.
- Ways to obtain the payment.
  1. You can choose the method of payment. Check/Deposit in the bank BANRURAL.[1]
  2. The payment will be made on two different dates. A first payment will be made BEFORE (Today or in 5 weeks), and a second payment will be made AFTER (In 5 or 9 weeks after the first payment).

Instructions for the CTB

Instructions for the first part of the experiment:

- General Instructions Part 1
  1. The first part consists of 24 questions.
  2. Each question has 6 options.
  3. For each question you should choose the option you prefer.
  4. When finished, we will randomly select one of the questions and we will pay the option you chose for that question.

[1] In initial sessions, participants had the option of deposits in their bank account or being paid via postdated checks. Since no particpants in these initial sessions opted for deposits, we did not offer this option in later sessions.

Example:[2]

TODAY I receive...	Q80	Q64	Q48	Q32	Q16	Q0
And in addition in 5 WEEKS I receive	Q0	Q20	Q40	Q60	Q80	Q100
	☐	☐	☐	☐	☐	☐
	Option 1	*Option 2*	*Option 3*	*Option 4*	*Option 5*	*Option 6*

- Each option is numbered on the bottom. Also, each option represents two quantities you can receive: a first payment (in this case, today), and also a second payment (in this case, in 5 weeks).
- The amounts in each option do not include the payment for participation.
- Option 1
  1. You would receive $Q80$ today.
  2. You would receive $Q0$ in 5 weeks.
- Option 2
  1. You would receive $Q64$ today.
  2. You would receive $Q20$ in 5 weeks.
- Option 3
  1. You would receive $Q48$ today.
  2. You would receive $Q40$ in 5 weeks.
- Option 4
  1. You would receive $Q32$ today.
  2. You would receive $Q60$ in 5 weeks.
- Option 5
  1. You would receive $Q16$ today.
  2. You would receive $Q80$ in 5 weeks.
- Option 6
  1. You would receive $Q0$ today.
  2. You would receive $Q100$ in 5 weeks.
- You must choose an option. Suppose you chose Option 3. Today, you will receive $Q48$, and also, in 5 weeks, you will receive $Q40$.
- Note that the options in this case allow you to receive up to $Q100$ if you receive it all in 5 weeks, or you can receive a maximum of $Q80$ if you receive everything today. There are also intermediate options. Today you can obtain $Q48$, and in 5 weeks $Q40$, for a total of $Q88$. The total increases as you want to receive more money in FIVE weeks.
- The dates of the first and the second payment vary with the question:
  1. Questions 1-6: In the first 6 Questions you choose between a FIRST payment to receive TODAY, and a SECOND payment to receive in FIVE WEEKS.
  2. Questions 7-12: In Questions 7-12 you choose between a FIRST payment to receive TODAY, and a SECOND payment to receive in NINE WEEKS.

[2] The example is modified according to the treatment.

3. Questions 13-18: In Questions 13-18 you choose between a FIRST payment to receive in FIVE WEEKS, and a SECOND payment to receive in TEN WEEKS.
4. Questions 19-24: In Questions 19-24 you choose between a FIRST payment to receive in FIVE WEEKS, and a SECOND payment to receive in FOURTEEN WEEKS.

- A drawing will be held to determine which question will be paid to you.[3] The 24 questions will not be paid to you. Only the option that is selected in the drawing.
- The dates when the FIRST and the SECOND payments are made will depend on the drawing.
- If question 14 is selected in the drawing, then the option you choose in question 14 will determine how much you will receive in FIVE WEEKS, and how much you will receive in TEN WEEKS.
- Your payment will be made through Banrural bank, via either deposits to your account or checks. In both cases, the money will be available on the two indicated dates (for your safety, all the dates are business days). The dates will be determinate by the question selected in the drawing.
- The payment for participating will be divided in two parts, and they will be paid on the dates when the payments of the question selected in the drawing are paid.
- So, what will be the amount you receive?
  1. You will receive the amount that the selected option indicates. Also, you will receive $Q50$ for your participation payment. This additional $Q50$ will be split in between the first and the second payment.
- At this point we will do a practice.
- The following three questions will not be among the questions entered into the drawing.
- During the practice, you can ask questions at any time.

[3] At this point a bingo cage with 24 balls inside is shown to the participants.
